# Supplementary material for: Rapid and inexpensive whole-genome sequencing of SARS-CoV-2 using 1200 bp tiled amplicons and Oxford Nanopore Rapid Barcoding
Source: Biol Methods Protoc. 2020 Jul 18;5(1):bpaa014. doi: 10.1093/biomethods/bpaa014 (PMC7454405; doi:10.1093/biomethods/bpaa014)
Supplement: bpaa014_Supplementary_Data [file bpaa014_supplementary_data.docx]

## Supplementary Table

**Table S1. Breakdown of time to complete each step of each protocol.**

| Steps* | 400 bp ARTIC Network LSK* | 1200 bp ARTIC Network RBK |
| --- | --- | --- |
| Reverse transcription | 1h 15m | 1h 15m |
| PCR | 3h | 3h |
| Clean up | 30m | 0 |
| End prep | 20m | 0 |
| Native barcode ligation | 1h | 0 |
| Adapter addition | 50m | 10m |
| Clean up | 20m | 0 |
| Loading | 10m | 10m |
| Total | 7h 25m | 4h 15m |
| **Approx hands on time:** | **2h 15m** | **25m** |

*Indicated times are as described by the Oxford Nanopore PCR tiling of COVID-19 virus protocol. LSK indicates Ligation Sequencing kit (SQK-LSK109), RBK indicates Rapid Barcode Kit (SQK-RBK004).
